# CTLA-4 and PD-1 combined blockade therapy for malignant melanoma brain metastases: mechanisms, challenges, and prospects

**DOI:** 10.3389/fimmu.2025.1629879

**Published:** 2025-07-01

**Authors:** Jia-Wen Wang, Ying-Fa Feng, Jia-Hui Liu

**Affiliations:** Department of Orthopedics, The Fourth Hospital of Hebei Medical University, Shijiazhuang, Hebei, China

**Keywords:** melanoma brain metastases, CTLA-4, PD-1, combined blockade therapy, immune checkpoint inhibitors, adverse event management, precision therapy

## Abstract

Malignant melanoma brain metastases (MBM) represent one of the deadliest complications of melanoma, with an incidence rate of 7.3%. Among patients with acral and mucosal melanoma, the cumulative 5-year incidence can reach 19.5%, accompanied by poor prognosis. The blood-brain barrier (BBB), an immunosuppressive central nervous system (CNS) microenvironment, and tumor immune evasion collectively limit the efficacy of traditional therapies. Cytotoxic T-lymphocyte-associated protein 4 (CTLA-4) and programmed cell death protein 1 (PD-1), as critical immune checkpoints, play pivotal roles in the progression of MBM. This study systematically analyzes the synergistic mechanisms, clinical outcomes, and challenges of CTLA-4 and PD-1 combined blockade therapy in MBM. The findings indicate that this combination therapy leverages a “priming and boosting” biological mechanism: CTLA-4 blockade broadens T-cell responses during the initial activation phase, while PD-1 blockade sustains T-cell activity during the effector phase, significantly improving intracranial response rates (46%, compared to 20% for monotherapy). Furthermore, the combination therapy increases the CD8+/Treg ratio and promotes memory CD8+ T-cell formation, enabling durable antitumor immune surveillance. However, challenges such as a 54% incidence rate of grade 3–4 adverse events and suboptimal therapeutic regimens remain. To address these issues, this study proposes a multi-tiered adverse event management system, personalized risk assessment models, and treatment optimization strategies based on real-time monitoring and dynamic adjustments. Future directions include developing precision stratified therapies based on immunogenomics, exploring multi-target synergistic approaches, and implementing intelligent adverse event prediction and management systems to maximize therapeutic efficacy and minimize toxicity, providing more effective treatment for MBM patients.

## 1 Introduction

Malignant melanoma is a highly aggressive skin cancer with a steadily increasing incidence. Among melanoma patients, brain metastases occur in approximately 7.3% of cases (1), with a cumulative 5-year incidence of up to 19.5% in acral and mucosal melanoma patients (2). Brain metastases progress rapidly, with 16.7% of patients diagnosed with CNS metastases during follow-up (3). Survival outcomes remain poor, particularly in male patients (4, 5). As one of the most lethal complications of melanoma, MBM has become a critical focus of oncological research.

MBM presents a unique immune microenvironment, posing significant challenges for immune therapy strategies. First, the BBB limits the penetration of most drugs into the CNS, reducing therapeutic efficacy (6). Second, the immunosuppressive microenvironment in the brain facilitates tumor immune evasion (7). Lastly, brain metastases often exhibit molecular and immune profiles distinct from their primary tumors (8). Traditional treatments such as surgical resection, whole-brain radiotherapy, and chemotherapy, while providing some benefit, are associated with limited efficacy and significant side effects, failing to improve long-term survival outcomes for patients (9). Therefore, developing more effective therapies for MBM is of paramount importance.

The advent of immune checkpoint inhibitors (ICIs) has revolutionized MBM treatment, particularly inhibitors targeting CTLA-4 and PD-1 (10). While these agents have shown efficacy in some MBM patients, the overall objective response rates remain suboptimal, with significant challenges related to primary and acquired resistance (11). To address these limitations, the combined blockade of CTLA-4 and PD-1, leveraging their distinct roles in different phases of T-cell activation, offers a promising therapeutic strategy with synergistic effects (12).

This paper focuses on the following key questions:

1. How does the immune microenvironment of brain metastases differ from primary tumors? What roles do CTLA-4 and PD-1 play in MBM progression? How do they modulate the tumor microenvironment and immune cell functions?
2. What clinical trial data support the application of combined therapy in MBM? How does it compare to monotherapy? How can immune-related adverse events (irAEs), particularly CNS toxicities, be effectively managed?
3. How can real-time biomarker monitoring be leveraged to optimize treatment dosing, sequencing, and duration to balance efficacy and toxicity? Under the guidance of immunogenomics, how might combination strategies involving CTLA-4/PD-1 inhibitors and small-molecule agents, next-generation immune checkpoint inhibitors, oncolytic viruses, cellular therapies, and vaccines reshape the therapeutic landscape of melanoma brain metastases?

## 2 Immune resistance and the basis of blockade therapy

A major challenge in the treatment of melanoma brain metastases (MBM) is the restrictive nature of the blood–brain barrier (BBB). Composed of tightly connected brain endothelial cells, a basal membrane, and astrocyte endfeet, the BBB effectively prevents most circulating immune cells and macromolecular drugs from entering the central nervous system (CNS) (6). Its integrity varies with metastatic lesion size: while micrometastases (<0.25 mm) typically maintain an intact BBB, larger lesions exhibit increased permeability yet still suffer from heterogeneous drug distribution (13). This spatial heterogeneity significantly impairs drug delivery and remains a key obstacle in MBM therapy. Melanoma cells further modulate BBB permeability by secreting vascular endothelial growth factor (VEGF) (14) and exosomes (15), promoting localized BBB disruption. However, single-agent immune checkpoint inhibitors (ICIs) often fail to efficiently penetrate the BBB. In contrast, combination therapy can reshape the immune microenvironment, enhance T cell activation and infiltration (16), and exploit BBB disruptions around metastatic foci (17), facilitating improved drug accumulation—an effect not consistently observed with monotherapy (16).

The MBM microenvironment poses additional barriers. TGF-β promotes immunosuppression by upregulating PD-1 and CTLA-4 on T cells, while IL-10 enhances CTLA-4-mediated inhibition by suppressing CD28 co-stimulation (18). Microglia interact with melanoma cells to promote malignant phenotypes (19), and astrocytes secrete proinflammatory cytokines such as IL-23, further driving tumor invasiveness (20). These interactions contribute to immune evasion and therapeutic resistance. However, dual checkpoint blockade can help overcome these mechanisms (21). For instance, PD-1/CTLA-4 combination therapy induces phenotypic changes in dendritic cells within brain metastases, increasing co-stimulatory molecules and proinflammatory cytokine expression, thereby mitigating resistance (22)—a benefit less evident with monotherapy (22).

PD-L1 upregulation also contributes to therapeutic resistance. Its expression in MBM is driven by multiple factors, including TGF-β, IFN-γ, and the EGFR–STAT3 pathway (23), leading to substantial intertumoral and intratumoral heterogeneity (24). Importantly, CTLA-4 and PD-1/PD-L1 pathways are mechanistically intertwined. CTLA-4 inhibition enhances T cell activation and IFN-γ secretion, which in turn upregulates PD-L1 expression on tumor cells, undermining CTLA-4 monotherapy efficacy (23). Dual blockade prevents this adaptive resistance, increases the CD8+/Treg ratio, and enhances therapeutic efficacy (25). Tumor cells can also evade immune surveillance by downregulating MHC expression and suppressing co-stimulatory signaling (26); yet, combination blockade effectively counters these escape mechanisms, restoring antitumor immunity (27).

## 3 Synergistic mechanisms of CTLA-4 and PD-1 combination therapy

**Figure 1:**
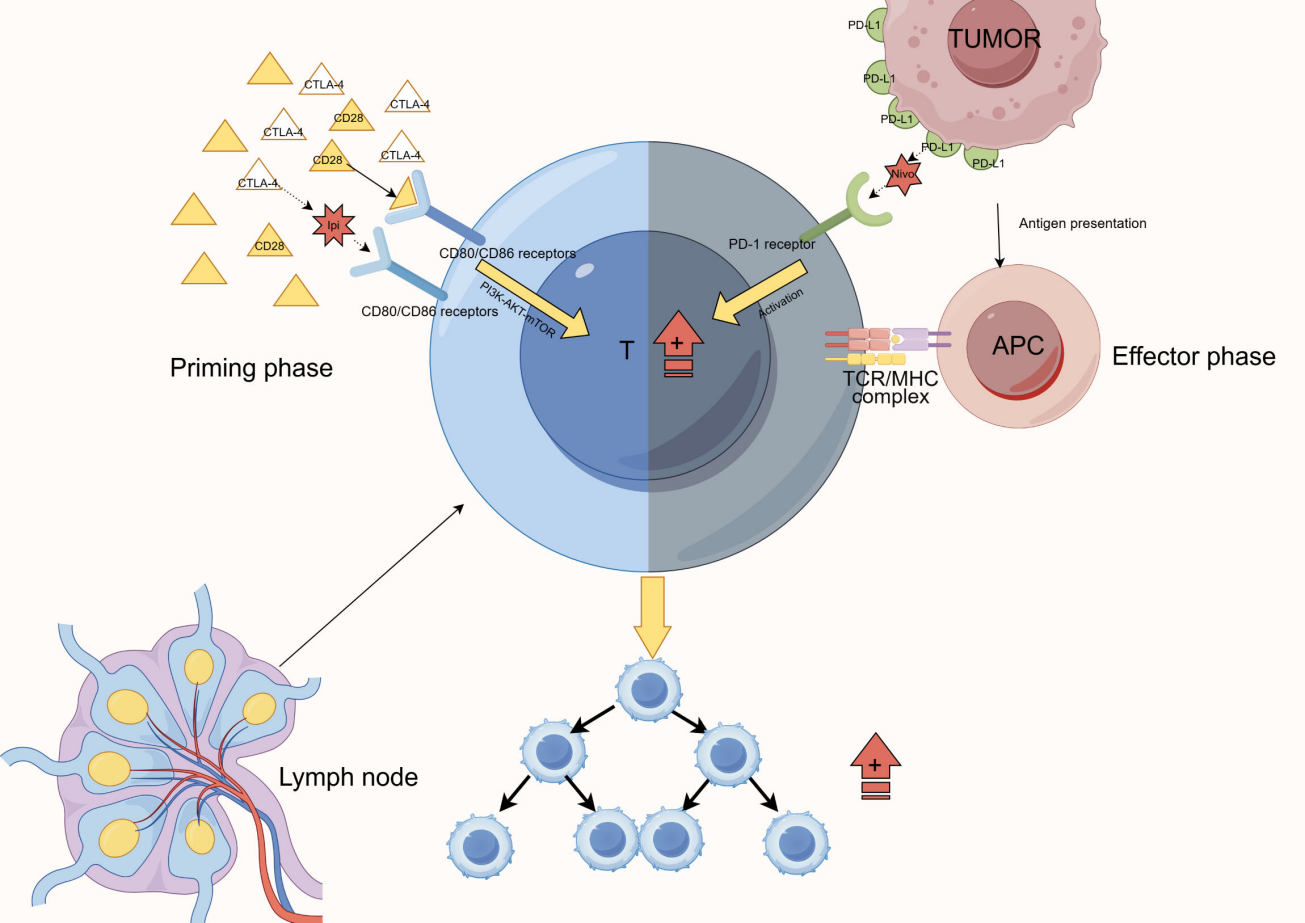
Mechanism of CTLA-4 and PD-1 blockade at different stages of T-cell activation. This figure illustrates the complementary actions of immune checkpoint blockade. Anti-CTLA-4 agents act at the initiation phase in lymph nodes, blocking CTLA-4-CD80/CD86 interactions to promote initial T-cell activation. In contrast, anti-PD-1 agents function at the effector phase in the tumor microenvironment, preventing PD-1-PD-L1 interactions to sustain T-cell functionality. Together, these agents achieve comprehensive regulation of the T-cell activation and effector processes, ultimately enhancing T-cell proliferation and function.

CTLA-4 and PD-1 dual blockade has demonstrated robust synergy in MBM treatment. **Table 1** outlines the mechanisms and clinical data of CTLA-4 blockade, **Table 2** summarizes the characteristics of PD-1 inhibitors, and **Table 3** highlights their synergistic therapeutic advantages. **Figure 1** illustrates their differential roles during T cell activation stages.

**Table 1:** Mechanism and data of CTLA-4 blockade.

Feature	Mechanism and Data	References
Action Phase	Early initiation phase of T-cell activation	(12)
Site of Action	T cell-APC contact points in peripheral lymphoid tissues	(12)
Molecular Mechanism	1. Blocks high-affinity binding of CTLA-4 to CD80/CD86 2. Reverses competitive inhibition of CD28 co-stimulatory signaling 3. Disrupts Tregs’ ability to downregulate CD80/CD86 expression on APCs	(69)
Effects on T Cells	1. Significantly lowers the T-cell activation threshold 2. Enables effective activation under weak antigen stimulation 3. Broadens the range of melanoma-reactive circulating CD8+ T cells	(29, 38)
Effects on Tregs	1. Selectively depletes infiltrating Tregs via ADCC mechanism 2. Weakens Tregs’ immunosuppressive functions	(30, 70)
Memory T-cell Effects	1. Promotes remodeling of memory T-cell subsets 2. Increases the proportion of central and effector memory T cells 3. Baseline memory T-cell levels correlate with therapeutic efficacy	(71, 72)
Significance in Brain Metastases	1. Generates large numbers of activated T cells 2. Upregulates chemokine receptors (e.g., CXCR3) and adhesion molecules 3. Enhances T-cell ability to cross the BBB	(6, 12)
Clinical Data	1. Combination with radiotherapy extends median survival to 21.3 months 2. Two-year survival rate increased to 47.2% 3. High expression of immune-related genes correlates with favorable outcomes	(73, 74)
Monotherapy Efficacy	1. Shows clinical benefit in patients with asymptomatic brain metastases 2. Limited overall efficacy as monotherapy 3. CTLA-4 blockade increases IFN-γ, which may upregulate PD-L1 expression	(31, 32, 75)

**Table 2:** Mechanism and data of PD-1 blockade.

Feature	Mechanism and Data	References
Action Phase	1. Effector phase of T-cell activation 2. Targets activated but potentially exhausted T cells	(12)
Site of Action	1. Tumor microenvironment (including brain metastases) 2. T cell-tumor cell contact points	(12)
Molecular Mechanism	1. Blocks PD-1 binding to PD-L1/PD-L2 2. Prevents functional exhaustion of T cells 3. Counteracts immune evasion due to PD-L1 upregulation	(23, 24, 33)
Effects on T Cells	1. Restores the cytotoxic function of antigen-stimulated T cells 2. Specifically targets tumor-specific T cells with high PD-1 expression	(34)
Effects on Cell Subsets	1. Expands specific CD8+ T-cell subsets 2. Enhances cytotoxic activity 3. Reduces Treg accumulation in tumors	(35, 42)
Efficacy in PD-L1 High Tumors	1. Effective against brain metastases with high PD-L1 expression 2. Blocks both cell surface and exosomal PD-L1 3. Limited monotherapy efficacy in PD-L1 high tumors	(24, 76)
Significance in Brain Metastases	1. Maintains functional capacity of T cells crossing the BBB 2. Upregulates receptors facilitating T-cell entry into tumor vasculature 3. Promotes T-cell migration to the brain	(12, 36)
Clinical Data	1. Monotherapy intracranial response rate ~20% 2. Combination with ipilimumab significantly improves response rates	(11, 37)
Monotherapy Limitations	Limited efficacy in symptomatic brain metastases	(32)

**Table 3:** Mechanism and data of CTLA-4 and PD-1 combination blockade.

Feature	Mechanism and Data	References
Phase Synergy	1. CTLA-4 acts at the initiation phase 2. PD-1 focuses on the effector phase 3. Enables comprehensive regulation of the entire T-cell activation process	(12)
Activation of T-cell Repertoire	1. CTLA-4 blockade broadens activation 2. PD-1 blockade sustains effector function 3. Improves both T-cell quantity and diversity	(38)
Mechanism for BBB Crossing	1. "Expand first, enhance later" biological mechanism 2. CTLA-4 blockade promotes peripheral T-cell expansion 3. PD-1 blockade upregulates T-cell entry receptors 4. Significantly improves T-cell migration to brain metastases	(12)
Addressing Tumor Antigen Heterogeneity	1. CTLA-4 blockade broadens T-cell response 2. Activates T cells targeting diverse tumor antigens 3. PD-1 blockade maintains functional activity of these clones	(39)
Diversity of Antigen-Specific T Cells	1. Activates low-frequency or low-affinity T-cell clones 2. NK cells synergistically regulate T-cell abundance 3. Prevents immune escape via "single-antigen loss"	(40, 77)
Blocking Immune Escape Pathways	1. Provides multi-layered protection from T-cell activation to effector function 2. PD-1 blockade expands CD8+ T-cell subsets 3. CTLA-4 blockade promotes Th1-like CD4+ T-cell expansion 4. Superior efficacy in PD-L1 high tumors compared to monotherapy	(35, 78)
CD8+/Treg Ratio	1. CTLA-4 blockade disrupts and depletes Tregs 2. PD-1 blockade maintains CD8+ T-cell activity 3. Reduces Treg accumulation 4. Significantly increases CD8+/Treg ratio in tumors	(12, 41, 42)
Memory T-cell Formation	1. Establishes a complete immune-promoting chain 2. Promotes memory CD8+ T-cell formation 3. Enhances tissue-resident memory T (TRM) cells 4. Optimizes memory T-cell quality	(12, 43)
Clinical Support	1. Combination therapy intracranial response rate 46%, far exceeding monotherapy (20%) 2. Response rate in asymptomatic patients reaches 54% 3. 20–30% of patients maintain long-term complete remission	(11, 37, 44)
Challenges and Limitations	1. Grade 3–4 adverse events occur in up to 54% of cases, significantly higher than monotherapy 2. Requires optimization of dosage, treatment sequence, and duration	(37, 79)

CTLA-4 is highly expressed on naïve and regulatory T cells (Tregs), where it competitively binds CD80/CD86 on antigen-presenting cells (APCs), thereby impeding full T cell activation (28). In Tregs, CTLA-4 also actively reduces CD80/CD86 expression on APCs, establishing a multilayered immunosuppressive network (28). Ipilimumab, a CTLA-4 inhibitor, lowers the T cell activation threshold and expands melanoma-reactive CD8+ T cell populations (29), while selectively depleting tumor-infiltrating Tregs via antibody-dependent cellular cytotoxicity (ADCC) (30). Although it can reprogram memory T cell subsets and enhance their BBB-penetrating ability (6), its efficacy as a monotherapy in brain metastases remains limited (31), likely due to CTLA-4 blockade-induced IFN-γ release, which upregulates PD-L1 expression and triggers adaptive resistance (32).

PD-1, in contrast, is predominantly expressed on activated T cells and plays a critical role at the tumor–T cell interface within brain metastases (12, 24). Blocking PD-1–PD-L1/PD-L2 interactions restores the cytotoxic function of antigen-experienced T cells, prevents exhaustion under chronic antigen exposure, and expands tumor-specific CD8+ T cell subsets (33–35). In MBM, PD-1 inhibitors preserve the effector function of T cells that have crossed the BBB and promote their migration into CNS lesions (36). However, the intracranial response rate to PD-1 monotherapy remains low—around 20%—especially in symptomatic MBM patients (32, 37).

Dual blockade of CTLA-4 and PD-1 overcomes these monotherapy limitations through a sequential synergy often described as “priming then sustaining.” CTLA-4 inhibition facilitates early T cell priming, increasing response breadth and clonal diversity, while PD-1 inhibition maintains effector T cell functionality during the response phase (12, 38). This strategy activates T cell clones against a broader range of tumor antigens, including low-affinity or low-frequency clones (39, 40). In MBM, this translates to improved BBB traversal and enhanced T cell infiltration into brain lesions (12). Furthermore, combination therapy significantly increases the CD8+/Treg ratio (41, 42), supports memory CD8+ T cell formation, and promotes tissue-resident memory T cells (TRMs), reinforcing long-term immune surveillance (43).

Clinical data corroborate these mechanistic insights: dual blockade yields intracranial response rates of up to 46%, markedly higher than monotherapy, and rates can reach 54% in asymptomatic patients. Moreover, 20–30% of patients achieve durable complete remission lasting multiple years (11, 37, 44).

## 4 Challenges

### 4.1 Adverse events

Dual blockade of CTLA-4 and PD-1 is associated with a high incidence (up to 54%) of grade 3–4 adverse events (AEs) (37), significantly exceeding those observed with monotherapies (**Table 3**). Mechanistically, this stems from the disruption of multiple immune tolerance checkpoints. CTLA-4 inhibition lowers the activation threshold of peripheral T cells, promoting polyclonal T cell expansion. Concurrently, PD-1 blockade enhances the effector function of these activated T cells, thereby increasing their cytotoxic potential against self-tissues. This “dual immune unleashing” effect compromises intrinsic immune tolerance, leading to a higher frequency and severity of immune-related adverse events (irAEs).

Studies have shown that combination therapy more frequently induces irAEs involving the skin, colon, endocrine organs, and liver (45). Neurologic irAEs (n-irAEs)—including meningitis, encephalitis, demyelinating syndromes, vasculitis, and peripheral neuropathies—are also more common with combined therapy (46). Additionally, immune checkpoint blockade alters the gut microbiome (47), which may further increase the likelihood of systemic autoimmune-like reactions (48).

Due to the severity of irAEs, systemic corticosteroids are often required for management (49). Although dual therapy offers improved intracranial response rates and prolonged survival in selected patients (37), treatment interruptions due to toxicity may compromise therapeutic efficacy and patient adherence (45). Notably, the increased incidence of neurologic irAEs has been associated with reduced overall survival (46). Therefore, effective AE management is not only essential for safety but also critical to optimizing clinical outcomes.

### 4.2 Challenges in treatment optimization

Another significant challenge is the suboptimal refinement of treatment regimens. Although the CTLA-4 and PD-1 combination demonstrates synergistic effects (9), current protocols lack personalization and fail to account for heterogeneity in disease presentation. Future studies must focus on tailoring regimens based on patient-specific factors to enhance therapeutic precision (50).

Furthermore, the temporal and functional differences between CTLA-4 and PD-1 regulation of T cell activity remain underutilized. While dual therapy improves intracranial responses and survival (9), it also increases irAE risk, including neurologic complications (46). Given that CTLA-4 and PD-1 modulate T cells at distinct stages of activation (12), rational treatment design should align with the drugs’ pharmacodynamic profiles rather than apply fixed combination regimens indiscriminately.

Interindividual variability in treatment response further underscores the need for precision medicine. Clinical markers such as tumor burden, lesion location, and serum lactate dehydrogenase (LDH) levels may guide treatment stratification (51). Notably, personalized strategies incorporating stereotactic radiosurgery (SRS) with PD-1 and CTLA-4 blockade have demonstrated durable intracranial control with manageable toxicity in MBM patients (50), suggesting a promising path toward maximizing efficacy while minimizing treatment-related harm.

## 5 Discussion

### 5.1 Strategies for managing adverse events

Given the high incidence of severe immune-related adverse events (irAEs), establishing a multi-tiered management framework is of paramount importance. Timely diagnosis and intervention are critical to prevent symptom escalation and the development of complications (52). This necessitates close monitoring and prompt action by experienced clinicians (49), alongside personalized treatment and management strategies tailored to individual patient factors, such as PD-L1 expression and medical history (52).

Due to the increased incidence of neurologic toxicities (53) and multi-organ irAEs associated with combination therapy (49), corticosteroids (e.g., methylprednisolone) are commonly employed as a first-line treatment. However, for severe or refractory cases, additional immunosuppressive agents may be required (49).

In parallel, the development of individualized risk assessment models has emerged as a promising direction to optimize therapy. Key biomarkers—such as MHC protein expression, CTLA-4 promoter methylation status, and immune cell profiling—can serve as predictive indicators of treatment response (54), potentially reducing therapy discontinuation and improving overall prognosis.

Moreover, optimizing combination dosing and scheduling is essential for mitigating toxicity. While dual checkpoint blockade offers substantial efficacy benefits (55), it is also associated with greater toxicity. Therefore, rational dose modulation and precise timing of drug administration are critical to achieve durable intracranial control with manageable toxicity levels in melanoma brain metastasis (MBM) patients (50).

### 5.2 Strategies for optimizing treatment regimens

To optimize therapeutic regimens, attention must be directed toward refining dosage, sequencing, and duration. First, the implementation of a “real-time monitoring–adaptive adjustment” strategy can be facilitated through dynamic biomarkers such as circulating tumor DNA (ctDNA) kinetics (56), the peripheral effector T cell to regulatory T cell ratio (57), and cytokine levels like IFN-γ (57). These indicators enable responsive dose adjustments during therapy.

Second, although concurrent CTLA-4 and PD-1 inhibition significantly improves outcomes in MBM (9), sequential regimens may further enhance efficacy and tolerability. Administering a CTLA-4 inhibitor as induction therapy followed by maintenance with PD-1 blockade can maximize treatment durability, improve quality of life, and balance efficacy with safety (51, 58).

Finally, tailoring dose intensity and treatment duration can strike a more favorable balance between therapeutic benefit and adverse effects, thereby achieving a more personalized and cost-effective treatment approach (59) that maximizes clinical benefit for patients.

### 5.3 Future perspectives

In the next 5–10 years, precision stratification systems guided by immunogenomic profiling are expected to become a prevailing trend. For instance, the density of CD16^+^ macrophages has been correlated with favorable responses to combination immunotherapy and may serve as a key biomarker for treatment stratification (60). Through immune microenvironment modulation, these approaches aim to enhance both the intracranial recruitment and peripheral expansion of CD8^+^ T cells, thereby improving the efficacy of combinatorial treatments (12). Consequently, the integration of CTLA-4/PD-1 inhibitors with small-molecule agents, antibodies, cellular therapies, and vaccines is emerging as a promising strategy for melanoma brain metastases (MBM) and warrants validation through prospective clinical trials incorporating stratified brain metastasis cohorts or dedicated MBM arms (see **Table 4**).

**Table 4:** Summary of mechanism-based CTLA-4/PD-1 combination strategies in melanoma brain metastases (MBM).

Category	Combination Type	Representative Regimen & Dosing/Phase	Key Mechanisms of Action*	Level of Evidence & Key Outcomes	References
Small Molecule Agents	MAPK-targeted triplet therapy	Atezolizumab 840 mg d1/15 + Vemurafenib 720 mg bid + Cobimetinib 60 mg d1–21 (TRICOTEL, BRAF^V600-mut cohort)	BRAF/MEK inhibition reduces tumor burden and upregulates MHC-I/tumor antigens; inhibits ERK-MAPK-CXCL8 axis → enhances BBB permeability & T cell infiltration; PD-L1 blockade restores effector T cell function	Phase II, n = 65; intracranial ORR 42%, median intracranial PFS 5.8 months; manageable safety profile	(61)
	Cell cycle regulation	Abemaciclib (CDK4/6i, 75 mg/kg, qd) + anti–PD-1 (mouse MBM model)	CDK4/6 inhibition ↑ MHC-I/tumor antigen & PD-L1 expression; suppresses Treg proliferation; promotes CD8^+^ T cell trafficking into brain; dual amplification of antigen presentation & immune checkpoint blockade	Preclinical: median OS increased by 65%; intracranial CD8^+^/Treg ratio doubled	(25)
	Epigenetic modulation	Vorinostat 400 mg qd + Pembrolizumab 200 mg q3w (Phase 1b); Class I HDACi + PD-1 blockade (melanoma in vitro/mouse model)	HDACi acetylates promoters → upregulates PD-L1 & MHC-I; induces CXCL9/10-mediated CD8^+^ recruitment; suppresses MDSCs; reprograms “cold” brain metastatic microenvironment	Phase Ib (multiple tumor types): DCR 67%; melanoma models: HDACi + PD-1 reduced tumor growth rate by 70%	(62, 63)
Antibody-Based	Next-generation checkpoint	Relatlimab (anti–LAG-3) + Nivolumab (RELATIVITY-020 MBM cohort)	LAG-3 co-expressed with PD-1 on exhausted T cells; dual blockade lifts two inhibitory pathways simultaneously, restoring intracranial CD8^+^ IFN-γ secretion	Phase I/IIa, n = 27; intracranial ORR 22.2%, CBR 63%; median OS 21.5 months (benefit observed even in PD-(L)1–resistant cases)	(64)
Oncolytic Virus	oHSV virotherapy	MSC-delivered oHSV + anti–PD-L1 (mouse MBM model)	oHSV induces immunogenic cell lysis; PD-L1 blockade eliminates adaptive resistance; ↑ IFN-γ^+^ CD8^+^ infiltration; >50% of mice achieved complete intracranial remission	Preclinical: >50% response rate; significant survival extension	(65)
Cell Therapy	TIL therapy	Lymphodepletion preconditioning + high-dose IL-2 + autologous TIL ± subsequent ICI	High-affinity TILs penetrate BBB and target micrometastases; CTLA-4/PD-1 blockade maintains long-term function	Retrospective, n = 17 untreated MBM patients: 7 intracranial CRs; combination with ICI/radiotherapy suggested for consolidation	(66)
Vaccine-Based	Immunomodulatory vaccine	IO102/IO103 (IDO/PD-L1 peptides) + Nivolumab (Phase 1/2, NCT03047928)	Vaccine induces IDO/PD-L1-specific CD4^+^/CD8^+^ T cells that eliminate immunosuppressive cells; synergistic with PD-1 blockade	Phase I/II, n = 30; systemic ORR 80%, CR 43% (MBM not included, but mechanism applicable)	(67)

This table summarizes current CTLA-4/PD-1-based combination strategies for melanoma brain metastases (MBM), encompassing small molecules, antibodies, oncolytic viruses, cell therapies, and vaccines. The levels of evidence range from preclinical studies to phase II clinical trials, underscoring the diverse mechanisms that enhance the central nervous system efficacy of immune checkpoint inhibitors.

MBM, melanoma brain metastases; ORR, objective response rate; PFS, progression-free survival; OS, overall survival; CBR, clinical benefit rate; CR, complete response; DCR, disease control rate; BBB, blood-brain barrier; TIL, tumor-infiltrating lymphocytes; ICI, immune checkpoint inhibitors; HDACi, histone deacetylase inhibitor; MDSC, myeloid-derived suppressor cell.

Most combination strategies remain in early-stage research and require larger, dedicated clinical trials to confirm long-term efficacy and safety in MBM patients.

Among small-molecule agents, BRAF/MEK inhibitors (e.g., Vemurafenib/Cobimetinib) combined with PD-L1 blockade (Atezolizumab) exert dual effects: rapid tumor burden reduction via MAPK pathway inhibition and enhanced T cell infiltration by upregulating tumor antigens and MHC-I expression, thus improving blood-brain barrier (BBB) permeability. This “inhibition-then-counterattack” strategy achieves an intracranial objective response rate (ORR) of up to 42%, with a median progression-free survival (PFS) of 5.8 months (61).

CDK4/6 inhibitors (e.g., Abemaciclib) enhance immunogenicity by increasing MHC-I and tumor antigen expression while suppressing regulatory T cell (Treg) proliferation, resulting in a higher CD8^+^/Treg ratio and improved synergy with PD-1 blockade, ultimately extending survival (25).

HDAC inhibitors (e.g., Vorinostat), via epigenetic modulation, can upregulate PD-L1 and MHC-I, promote T cell recruitment, and suppress myeloid-derived suppressor cells (MDSCs). When combined with PD-1 inhibitors (e.g., Pembrolizumab), these agents help reprogram the “cold” brain metastasis microenvironment, inhibit tumor progression, and achieve high disease control rates (62, 63).

For immune checkpoint antibody-based therapies, co-targeting LAG-3 alongside CTLA-4 and PD-1 blockade may enhance efficacy while reducing toxicity (52). Dual inhibition of LAG-3 and PD-1 pathways effectively restores CD8^+^ IFN-γ production within the CNS (64), thereby improving intracranial T cell activation and migration (12). This multi-target synergistic blockade is a promising strategy to maximize clinical benefit while minimizing immune-related adverse events (irAEs) (48).

Oncolytic virus therapy utilizing mesenchymal stem cell (MSC)-delivered oHSV mediates immunogenic tumor cell lysis and releases damage/pathogen-associated molecular patterns (DAMPs/PAMPs). This activates type I interferon responses, and when combined with PD-L1 antibodies, can overcome adaptive immune resistance. In preclinical models, this strategy resulted in complete intracranial remission in the majority of treated mice (65).

In the realm of cellular therapy, tumor-infiltrating lymphocytes (TILs) infused after lymphodepleting preconditioning and supported by high-dose IL-2 can penetrate the BBB and eradicate micrometastases. Subsequent maintenance with CTLA-4/PD-1 inhibitors promotes persistence and durable responses. Notably, in a trial of 17 untreated MBM patients, 7 achieved long-term complete remission with TIL therapy alone, highlighting the potential for broader benefit when combined with ICIs (66).

With respect to immunomodulatory vaccines, the IDO/PD-L1 peptide vaccine (IO102/IO103) in combination with Nivolumab can elicit IDO/PD-L1-specific CD4^+^ and CD8^+^ T cell responses, targeting and eliminating immunosuppressive IDO^+^/PD-L1^+^ cells and converting the tumor microenvironment from “cold” to “hot.” This strategy achieved an ORR of up to 80% and a CR rate of 43%, offering a novel immunotherapeutic avenue for brain metastases (67).

Finally, the development of AI-driven systems for adverse event prediction and management may significantly reduce irAEs (68), facilitating safer and broader clinical implementation of these advanced combination strategies.

#### 5.3.1 Permission to reuse and copyright

Permission must be obtained for use of copyrighted material from other sources (including the web). Please note that it is compulsory to follow figure instructions.

## Data Availability

The original contributions presented in the study are included in the article/supplementary material. Further inquiries can be directed to the corresponding author.
